# Interplay of Luminophores and Photoinitiators during Synthesis of Bulk and Patterned Luminescent Photopolymer Blends

**DOI:** 10.1021/acsapm.4c00484

**Published:** 2024-05-27

**Authors:** Helen Tunstall-García, Takashi Lawson, Kathryn A. Benincasa, Andrew W. Prentice, Kalaichelvi Saravanamuttu, Rachel C. Evans

**Affiliations:** † Department of Materials Science and Metallurgy, University of Cambridge, Cambridge CB3 0FS, U.K.; ‡ Department of Chemistry and Chemical Biology, McMaster University, Hamilton L8S 4M1, Canada; § School of Engineering & Physical Sciences, Heriot-Watt University, Edinburgh EH14 4AS, U.K.

**Keywords:** photopolymerization, acrylates, methacrylates, epoxides, siloxanes, luminophores, waveguide-encoded lattices, self-induced waveguides

## Abstract

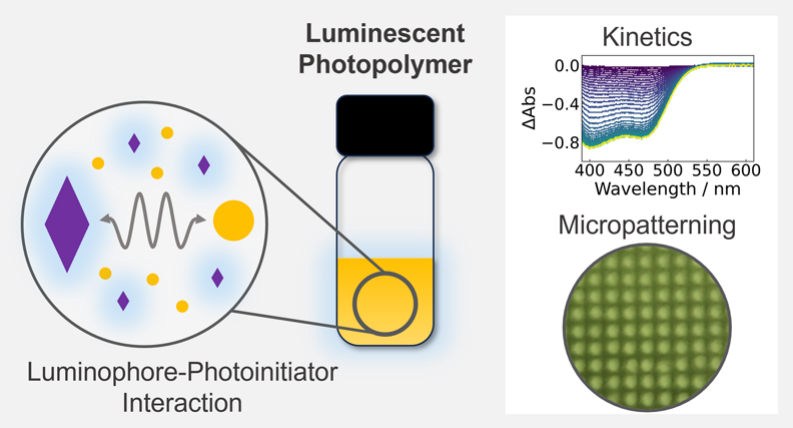

Four-dimensional printing with embedded photoluminescence is emerging as an exciting area in additive manufacturing. Slim polymer films patterned with three-dimensional lattices of multimode cylindrical waveguides (waveguide-encoded lattices, WELs) with enhanced fields of view can be fabricated by localizing light as self-trapped beams within a photopolymerizable formulation. Luminescent WELs have potential applications as solar cell coatings and smart planar optical components. However, as luminophore-photoinitiator interactions are expected to change the photopolymerization kinetics, the design of robust luminescent photopolymer sols is nontrivial. Here, we use model photopolymer systems based on methacrylate-siloxane and epoxide homopolymers and their blends to investigate the influence of the luminophore Lumogen Violet (**LV**) on the photolysis kinetics of the Omnirad 784 photoinitiator through UV–vis absorbance spectroscopy. Initial rate analysis with different bulk polymers reveals differences in the pseudo-first-order rate constants in the absence and presence of **LV**, with a notable increase (∼40%) in the photolysis rate for the 1:1 blend. Fluorescence quenching studies, coupled with density functional theory calculations, establish that these differences arise due to electron transfer from the photoexcited **LV** to the ground-state photoinitiator molecules. We also demonstrate an in situ UV–vis absorbance technique that enables real-time monitoring of both waveguide formation and photoinitiator consumption during the fabrication of WELs. The in situ photolysis kinetics confirm that **LV**-photoinitiator interactions also influence the photopolymerization process during WEL formation. Our findings show that luminophores play a noninnocent role in photopolymerization and highlight the necessity for both careful consideration of the photopolymer formulation and a real-time monitoring approach to enable the fabrication of high-quality micropatterned luminescent polymeric films.

## Introduction

Photopolymerization is an underpinning process for next-generation fabrication technologies such as additive manufacturing and three-dimensional (3D) printing,^1−3^ which find diverse applications ranging from rapid prototyping^4,5^ and microfluidics,^6,7^ to optical computing^8^ and dentistry.^9^ In photopolymerization, the covalent assembly of liquid-state monomers into polymer chains or networks is driven by excited-state reactive species that form upon irradiation of the photocurable medium with light. Light is the ideal trigger since it can be applied remotely with discrete spatial and temporal control to embed specific patterns within the finished material.^1,10^ Photopolymerization reactions typically occur through either a free-radical or a cationic mechanism, with methacrylate and epoxy resins being common precursors, respectively.^2^ In both cases, a photoinitiator is required for the reaction to take place upon irradiation, either through direct initiation, where a photoinitiator generates free radicals or cations upon absorption of light through photolysis,^11^ or indirect initiation, where a secondary co-initiating species is required. In some cases, a single photoinitiator can produce both radicals and cations to enable the formation of interpenetrating polymer networks and blends.^12^

Recently, four-dimensional (4D) printing has emerged as an exciting area to manufacture designs with additional functionality, for example, by including programmable materials with a stimuli-response^13−15^ or a luminophore to embed photoluminescence.^16−18^ Spatial patterning can be achieved by localizing the irradiation beam within the photocurable formulation during polymerization. For example, a tightly focused laser beam can be used in direct laser writing to form structures with sub-100 nm resolution in depletion mode.^19^ However, micron-scale resolution can readily be achieved at a much lower cost using incoherent light sources such as light-emitting diodes (LEDs) in conjunction with a photomask of well-defined pattern.^20,21^ The latter approach has been effectively harnessed to design a class of photonic material known as waveguide-encoded lattices (WELs).^22−24^ WELs form under nonlinear conditions during photopolymerization, where incoherent light beams undergo self-trapping–a process where the natural divergence of light is suppressed.^25,26^ The use of a mask and lenses to control the incident light pattern in the photocurable medium leads to the formation of microscopic arrays of self-trapped light beams that induce photopolymerization along their propagation path. The result is a patterned array of cylindrical waveguide channels of high refractive index formed by the beam path embedded within a matrix of lower refractive index. WELs have been shown to form in various photopolymerizable media, including methacrylate-siloxanes^23,27^ and epoxides.^22,24^ Notably, the waveguide array pattern is easily tunable, which has been exploited to achieve impressive fields of view of light capture of up to 115°,^22^ with potential applications as coatings for solar cells,^28^ and for the control of broad beams of light.^23^ However, while WELs present significant opportunities for enhanced light capture and control, they are passive films that do not enable modulation of incoming light.

Recently, active luminescent WELs, herein referred to as LWELs, have also been demonstrated based on a binary photopolymer blend of the acrylate-based commercially available Norland Optical Adhesive 65 (NOA 65) and an epoxide-terminated polydimethylsiloxane (PDMS) oligomer.^29^ Camphorquinone (CQ) was used as the free-radical photoinitiator, together with (4-octyloxyphenyl)phenyliodonium hexafluoroantimonate (OPPI) as co-initiator, to drive the epoxide end-group photopolymerization of PDMS. The luminophore fluorescein o,o′-dimethacrylate was also included in the blend (assumed to graft to NOA 65), which enables spectral conversion of incident UV-blue light to green photoluminescence in the final material. Previous studies by the same group suggest that the native polymer blend phase separates, with the acrylate component residing in the WEL channels and the PDMS forming the bulk, leading to increased refractive index contrast.^30^ In the LWEL system, the addition of fluorescein increases this refractive index contrast, likely due to increased phase separation or a greater refractive index across the sample, especially along the waveguide channels.^29^ This is perhaps unsurprising since it is well known that absorbance spectrum overlap between the photosensitizer and embedded luminophore (as is the case for CQ and fluorescein) affects both the cure depth and degree of photopolymerization.^18,31^ For example, detailed studies by Lalevée and co-workers demonstrated that fluorescent dyes from the naphthalimide family could influence the photoinitiation of methacrylates, acrylates, and their blend systems using both free-radical and cationic polymerization mechanisms. This, in turn, affected both the integrity and mechanical properties of 3D-printed structures; however, the fluorescence properties were not investigated in detail.^16^

Given these limitations, herein we investigate the photopolymerization kinetics of bulk and micropatterned (i.e., WELs) monoliths formed by homopolymers and their blends with and without a luminophore. Our objectives are to understand (i) how luminophore-photoinitiator interactions affect the photolysis rate and (ii) whether this affects the emission properties of the final material. To achieve this, we have designed a model blend based on methacrylate-siloxane and epoxy-terminated PDMS precursors in which both components can be photoinitiated by the same primary sensitizer ([(bis(η^5^-cyclopentadienyl) bis(2,6-difluoro-3-(1H-pyrrole-yl)-phenyl) titanium(IV)], herein referred to as Omnirad 784) to reduce the complexity of the system and minimize absorbance overlap. Time-resolved fluorescence quenching experiments are combined with density functional theory (DFT) calculations to understand electronic interactions between the luminophore and photoinitiator. Finally, we demonstrate how in situ optical spectroscopy can be used to monitor micropatterning during photopolymerization and show how photobleaching of the photoinitiator affects the final photoluminescence properties.

## Results and Discussion

### Design of the Luminescent Polymer System

**Figure 1:**
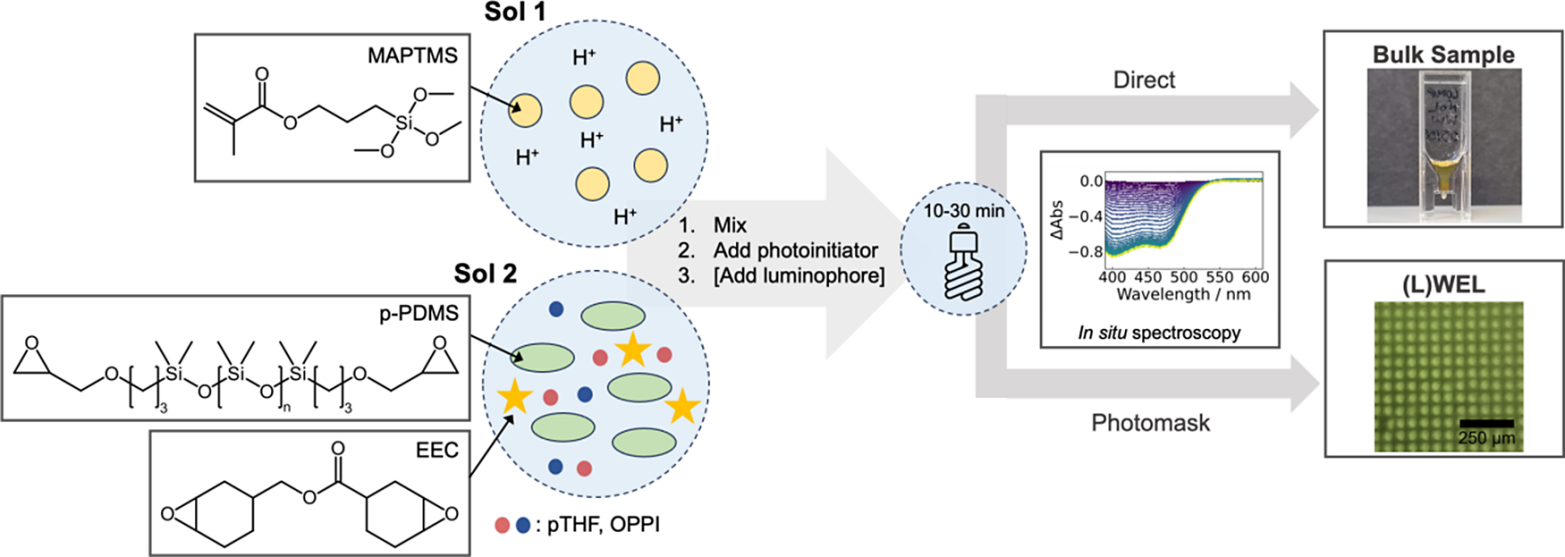
Schematic representation of bulk sample and (L)WEL fabrication by photopolymerization. Sol 1 consisted of the methacrylate-substituted siloxane precursor, MAPTMS, and HCl (0.05 M). Sol 2 contains the epoxide component system based on p-PDMS and EEC, pTHF (hydrogen donor), and OPPI (co-initiator). The sols are either treated individually or mixed in a 1:1 blend. The photoinitiator (Omnirad 784) and luminophore (Lumogen Violet) are added to the sols before irradiation with white light (halogen or LED). Direct irradiation results in bulk polymer samples, while the presence of a photomask leads to micropatterned (L)WEL samples. For (L)WEL samples, the kinetics upon photoirradiation are followed by in situ UV–vis absorbance spectroscopy.

Figure 1 presents a schematic illustration of the precursor homopolymers, the polymerization process, and representative photographs of both bulk and micropatterned (WEL) samples. In brief, the precursor polymer sols are prepared and mixed (if making a blend) before the photoinitiator and luminophore (if using) are added. The sol is then exposed to white light (halogen or LED), either directly for bulk samples or through a photomask for WEL samples. Full experimental details can be found in the Supporting Information.

The methacrylate-substituted siloxane system (Sol 1, Figure 1) has previously been used to fabricate WELs.^23^ These photopolymerizable sols were prepared in two steps. First, acid-catalyzed hydrolysis and polycondensation of trimethoxysilyl groups of precursor molecules generate the poly(siloxane) backbone. Subsequent exposure of the sol to visible light leads to photoinduced free-radical polymerization of methacrylate substituents (Scheme S1, Supporting Information). The resultant WELs show a refractive index change (Δ*n*) of 0.006 at the waveguide channel interface, leading to efficient light guiding through the channels.^22^ However, the organosiloxane films are extremely brittle and difficult to remove from the sample holder.^27^

The photosensitized epoxide component system (Sol 2, Figure 1) has also produced WELs.^22,24^ In this system, cationic ring-opening polymerization (ROP) occurs between the terminal epoxide groups on the precursors (3,4-epoxycyclohexylmethyl 3,4–epoxycyclohexane carboxylate (EEC) and epoxypropoxypropyl-terminated polydimethylsiloxane (p-PDMS)) upon light exposure in the presence of the photoinitiator.^32^ EEC was selected for its ability to undergo photopatterning by self-trapping and waveguide formation upon visible-light polymerization.^33^ In this system, a hydrogen donor (poly(tetrahydrofuran), pTHF) and co-initiator (4-octyloxyphenyl phenyliodonium hexafluoroantimonate, OPPI) are required to drive the reaction (Scheme S2, Supporting Information). pTHF was chosen over other hydrogen donors as it retains high polymerization rates while improving the mechanical and thermal properties of the resulting material. The refractive index change (Δ*n* ∼ 0.001) in this system is slightly lower, but the resulting films are thermochemically robust and free standing.^22^

Based on the outlined properties of the individual homopolymers, we postulated that a blend of methacrylate-substituted siloxane (**Acr-Sil**) and epoxide (**Epo**) should result in films that combine the best of both systems. Screening studies demonstrated that well-defined WELs could be formed in an **Acr-Sil: Epo** blend ratios of both 1:1 and 3:2, resulting in free-standing films with good mechanical integrity (Figure 1).

**Figure 2:**
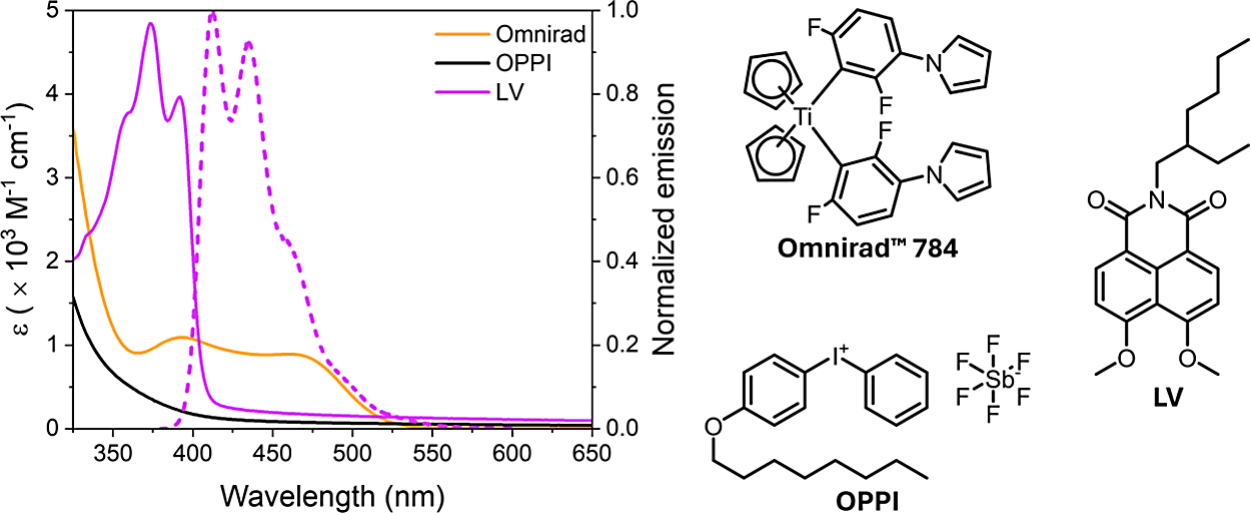
UV–vis absorbance spectra and chemical structures of Omnirad 784, OPPI, and Lumogen Violet (**LV**) in toluene (solid lines). The fluorescence spectrum of **LV** (λ_exc_ = 375 nm) is superimposed for comparison (dashed line).

The choice of luminophore and photoinitiator was considered in tandem to allow us to investigate how electronic interactions between these two components influence polymerization kinetics. In previous studies on WELs, free-radical polymerization of **Acr-Sil** was photoinitiated by Omnirad 784,^23^ while cationic ROP of **Epo** was achieved using CQ as the photoinitiator with OPPI as a co-initiator.^22,24^ However, a single photoinitiator is desirable to simplify the study of the reaction kinetics. Omnirad 784 was selected over CQ as it photolyzes during the initiation of photopolymerization, providing a convenient handle to monitor the photoinitiation kinetics. Test studies demonstrated that cationic ROP of **Epo** could be successfully photoinitiated using Omnirad 784 in conjunction with OPPI to produce WELs (see Figure S2, Supporting Information). We note that the Omnirad 784 co-initiator is required to ensure the reaction proceeds under white light rather than UV irradiation so that a single and safer light source can be used to drive the polymerization of **Acr-Sil** and **Epo** sols.^34^ We chose Lumogen Violet (**LV**) as the luminophore due to its good photostability and high photoluminescence quantum yield (PLQY ∼ 99% in toluene^35^). Owing to the near unity PLQY, we expect no formation of triplet excited states, which could result in undesired side reactions. Figure 2 overlays the absorbance spectra of Omnirad 784, OPPI, and **LV**. While Omnirad 784, OPPI, and **LV** exhibit different absorbance profiles, they overlap significantly from 300 to 410 nm, which coincides with two key emission lines (366 and 405 nm) from the halogen lamp (Figure S3, Supporting Information). The absorbance overlap is less significant with the white LED (negligible emission from 300 to 400 nm), resulting in a greater proportion of the total incident photons (with wavelengths above 400 nm) being absorbed by Omnirad 784. As such, the fraction of photons absorbed by each component will depend strongly on the wavelength-dependence of the molar absorbance coefficient (ε) and the spectral profile of the lamp, along with the relative loading of photoinitiator, photosensitizer, and **LV** in the precursor sols (wt %).

### Photolysis Kinetics within Bulk Homopolymers and Polymer Blends

**Figure 3:**
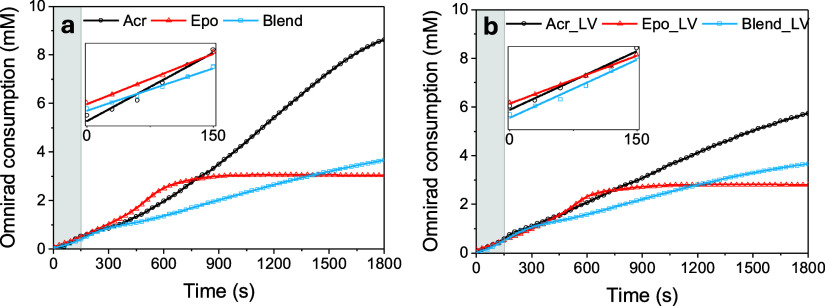
Initial rate analysis of photoinitiator consumption during photopolymerization of bulk **Acr-Sil**, **Epo,** and **Blend 1:1** sols. The molar consumption of Omnirad 784 with time under halogen lamp irradiation (0.63 mW cm^–2^) was monitored by UV–vis absorbance spectroscopy for sols (a) without **LV** and (b) with **LV** (0.02 wt %). The gray shaded regions (0–150 s) indicate the linear region where an initial rate fit to a pseudo-zero-order rate equation was performed (insets).

The photoinitiation process, whereby light is absorbed by Omnirad 784 to generate free radicals, has previously been studied using UV–vis absorbance spectroscopy to extract rate constants.^36^ We used a similar approach to investigate this process in the bulk homopolymers and their blends by monitoring the molar consumption of Omnirad 784 under halogen lamp excitation through UV–vis absorbance spectroscopy (Figure 3, see Figures S4 and S5, Supporting Information for further details). We used a blend composition of 1 part **Acr-Sil** to 1 part **Epo** by volume, herein referred to as **Blend 1:1**. The type of sol has a marked effect on the rate and degree of Omnirad 784 consumption: for **Epo** sols, a plateau consumption of ∼75% is observed after 1800 s, while for **Acr-Sil** and **Blend 1:1** sols, no consumption plateau was reached during this same period of time. We attribute this lack of plateau to the higher starting concentrations of Omnirad 784 in **Acr-Sil** and **Blend 1:1** (10 mM in **Acr-Sil** vs 7 mM in **Blend 1:1** vs 4 mM in **Epo** sol). The initial consumption of Omnirad 784 appears slightly above 0 mM (<0.1 mM) at 0 s due to a small (<1 s) delay between collecting a reference spectrum, starting the spectral measurement, and activating the halogen lamp source.

The initial rate of Omnirad 784 consumption was fitted to a pseudo-zero-order rate law to quantify and compare the photolysis rate of the photoinitiator in different sols over the first 150 s of irradiation. Over this period, linear consumption is observed for all samples (Figure 3a, Table 1), indicating that the initiation process is rate-limited by the photon flux rather than the photoinitiator concentration (Figure S6, Supporting Information). The initial rate was slightly higher for the **Acr-Sil** sol (3.84 ± 0.33 μM s^–1^) compared to that of the **Epo** sol (2.83 ± 0.10 μM s^–1^). The difference in photolysis behavior between **Acr-Sil** and **Epo** sols is attributed to a higher percentage of photon absorption by Omnirad 784 in **Acr-Sil**, in which there is no competition with OPPI. We note that a control experiment showed that the polymerization of **Epo** does not proceed in the absence of OPPI. Furthermore, the two systems undergo different polymerization mechanisms. It has also been previously reported that Omnirad 784 photolysis occurs via several intermediates within **Epo** sols, which could explain the slower kinetics.^36^ The lower rate in **Epo** could also originate from its more acidic environment (pTHF is a proton donor), which will affect the protonation of Omnirad 784 and subsequent photolysis products.^37^

**Table 1:** Photolysis Parameters Determined for Omnirad 784 during Photopolymerization of **Acr-Sil**, **Epo,** and **Blend 1:1** Sols, with and without **LV**, under Irradiation with the Halogen Lamp (0.63 mW cm^–2^)

**sample**	***k***^a^**(×10**^**–6**^M s^–1^**)**	***R***^**2**^^b^	**degree of consumption**(*t* = 150 **s, %)**^c^	**degree of consumption** (*t* = 1800 **s, %)**^d^	**photoinitiation yield**^e^
**Acr-Sil**	3.84 ± 0.33	0.971	5.4	86.3	2.89 ± 0.25
**Acr-Sil LV**	3.27 ± 0.21	0.984	5.8	57.3	2.46 ± 0.16
**Epo**	2.83 ± 0.10	0.995	13.1	76.2	2.46 ± 0.09
**Epo LV**	2.67 ± 0.06	0.998	13.2	70.1	2.32 ± 0.05
**Blend**1:1	2.38 ± 0.14	0.987	5.7	52.4	2.11 ± 0.12
**Blend**1:1 **LV**	3.25 ± 0.23	0.980	7.3	52.4	2.88 ± 0.20

a Pseudo-zero-order rate constant and

b goodness of fit for initial rates fit in linear region (0–150 s).

c Normalized degree of consumption at 150 s.

d Normalized degree of consumption at 1800 s.

e Initiation yield–the number of moles of Omnirad 784 consumed per mole of photons absorbed by Omnirad 784. See Section 7.4, Supporting Information for calculation details.

A slower rate is observed for the **Blend 1:1** sol (2.38 ± 0.14 μM s^–1^) than in homopolymer sols, contradicting the trend observed in the percentage of photon absorption by Omnirad 784, whereby we would expect the **Blend 1:1** rate to lie between that of **Acr-Sil** and **Epo**. The photolysis kinetics of the blend cannot be easily deconvoluted into independent acrylate and epoxide elements, suggesting that there is some interaction between the photoinitiation processes for **Acr-Sil** and **Epo**. This highlights a challenge in fabricating new photopolymers–namely, the two photopolymer components in a blend can interact and affect the resulting photopolymerization kinetics.

Interestingly, the initial rate deviates for the sols upon the addition of **LV** (Figure 3b), and the effect varies with sol composition. For **Acr-Sil**, Omnirad 784 consumption decreases slightly (to 3.27 ± 0.21 μM s^–1^), indicative of a slower photolysis rate, while for **Epo** sols, the rate is unaffected. Furthermore, the degree of consumption at *t* = 1800 s in the **Acr-Sil LV** reveals a significant decrease in photolysis (86.3% cf. 57.3%), while the **Blend 1:1** and **Epo** sols remain unchanged. Our calculations show that adding a luminophore results in competition for photon absorption, with 34% of photons absorbed by **LV** in both sols (Table S1, Supporting Information). Photon competition explains the slower photolysis rate of Omnirad 784 in **Acr-Sil** sols upon adding **LV**. However, in **Epo** sols, a decrease in the photolysis rate is not observed, suggesting that a photon absorbed by **LV** may also lead to the consumption of an Omnirad 784 molecule.

A considerable increase in the photolysis rate was observed for **Blend 1:1** (from 2.38 ± 0.14 to 3.25 ± 0.23 μM s^–1^) upon the addition of **LV**, which was unexpected. This increase results in a concomitant rise in the photoinitiation yield (2.11 ± 0.12–2.88 ± 0.20) and a higher degree of consumption at 150 s (5.7–7.3%) for the **Blend 1:1 LV**. This demonstrates the anomalous behavior in the **Blend 1:1** system, which differs from that observed in **Acr-Sil** and **Epo** sols and can no longer be described solely by photon competition, suggesting a secondary photolysis mechanism.

**Figure 4:**
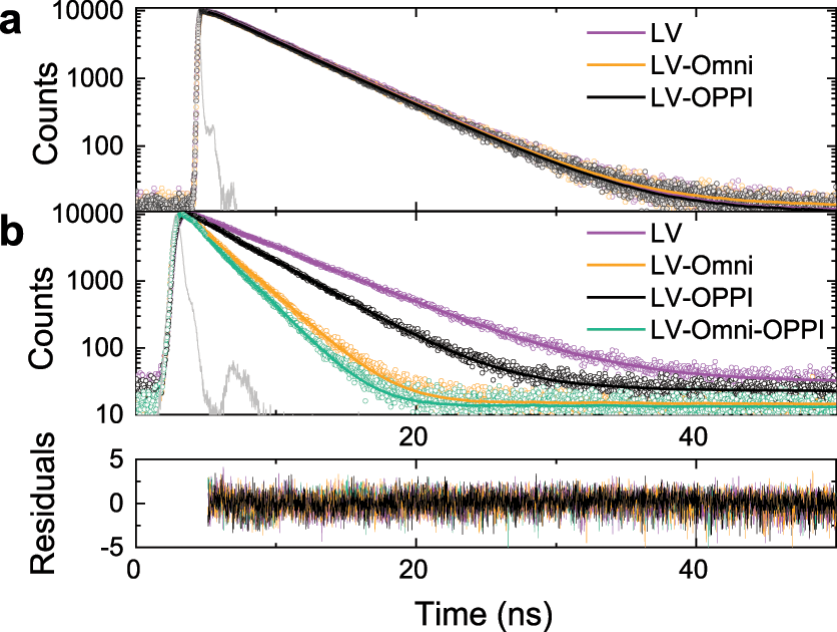
Effect of photoinitiators on the fluorescence decay kinetics of **LV** in toluene (λ_exc_ = 375 nm, λ_em_ = 430 nm). (a) Low-concentration (5 μM **LV**) decay curves showing no quenching effect upon the addition of Omnirad 784 (200 μM) or OPPI (550 μM). (b) High concentration (300 μM **LV**) photoluminescence decay curves showing a decrease in lifetime upon the addition of OPPI (30 mM), Omnirad 784 (10 mM), and both photoinitiators simultaneously (10 and 30 mM, respectively). The solid lines show a single exponential fit to the decay curves. The goodness of fit is demonstrated by the residuals in the bottom panel.

The potential interaction between **LV**, Omnirad 784, and OPPI was explored by time-resolved fluorescence quenching. All fluorescence decay curves were obtained by using toluene as a solvent and could be fitted to a single exponential. At low luminophore concentrations (5 μM in toluene), the fluorescence lifetime of **LV** (τ = 4.6 ns) remains unchanged upon the addition of Omnirad 784 (200 μM, τ = 4.6 ns) or OPPI (550 μM, τ = 4.6 ns) at similar molar ratios as those used in the polymer sols (Figure 4a, Table S2, Supporting Information). This indicated the absence of fluorescence quenching at low luminophore concentrations. However, at a higher **LV** concentration (300 μM in toluene), comparable to that used in polymer sols, a clear decrease in the fluorescence lifetime (τ = 5.1 ns) was observed in the presence of either Omnirad 784 (10 mM, τ = 2.3 ns) or OPPI (30 mM, τ = 3.7 ns), (Figure 4b, Table S2, Supporting Information). This indicates that the Omnirad 784 photoinitiator quenches the **LV** fluorescence more effectively than the OPPI co-initiator, despite being present at significantly lower concentrations than OPPI. A solution containing both Omnirad 784 (10 mM) and OPPI (30 mM) shows a marginal increase in quenching, as shown by the fluorescence lifetime of τ = 2.0 ns.

We attribute this fluorescence quenching and the enhanced photoinitiation kinetics observed primarily to electron transfer from **LV** to Omnirad 784, as supported by DFT simulations (Section 7.6, Supporting Information). The DFT results demonstrate that reactions in which an electron is transferred from **LV** to Omnirad 784 are preferred in all cases. While the free energies obtained for the adiabatic species suggest all electron transfer reactions to be slightly endergonic (Table S3, Supporting Information), consideration of the energy available upon photoexcitation (Table S4, Supporting Information) suggests that electron transfer from the photoexcited **LV** to the ground-state Omnirad 784 species is the lowest energy reaction and a feasible pathway, with a free energy of 1.63 kJ/mol falling within the limits of error in the calculations. Furthermore, all the calculations were performed at a temperature of 298 K, and considering the increase in local temperature (up to ∼150 °C)^34^ caused by photopolymerization, we expect the endergonicity of the electron transfer reactions to be further reduced. Finally, analysis of the HOMO–LUMO orbital energies further supports electron transfer from the photoexcited **LV** to Omnirad 784 as the most favorable mechanism (Figure S8, Supporting Information). As such, we postulate that at high concentrations of both **LV** and Omnirad 784, the intermolecular distance between both species is reduced such that electron transfer from the excited-state luminophore donor to the ground-state photoinitiator acceptor is preferred.^38^ The dependence on the intermolecular distance between the two species explains the lack of fluorescence quenching at low concentrations of **LV**. For the quenching mechanism of OPPI, any electron transfer process must involve its ground state since OPPI does not absorb at the excitation wavelength used (375 nm). Electron donation to ground-state iodonium salts has been frequently reported,^39^ triggering degradation of the phenyliodonium fragment into Ph· and PhI. DFT calculations on the excited state and the one-electron-reduced and -oxidized species of OPPI corroborate this mechanism. Analysis of the HOMO–LUMO energies (Figure S8, Supporting Information) shows that electron transfer from the photoexcited **LV** to OPPI is possible, with a greater thermodynamic driving force than Omnirad 784. Therefore, based on the thermodynamic calculations and the concentrations used, the origin of the increased quenching observed for Omnirad 784 (Figure 4) most likely lies in the kinetics of the electron transfer process.

In summary, luminophores play a noninnocent role when incorporated into photopolymer sols, often having a complex effect on the photopolymerization kinetics, as exemplified by Omnirad 784 and Lumogen Violet. Our studies point toward electron transfer between the photoinitiator and luminophore as the cause. This means that the optimized protocol for photopolymerization without a luminophore may not necessarily translate when a luminophore is incorporated. Therefore, monitoring the reaction in situ to reveal any differences is prudent.

### Photolysis Kinetics within Patterned Polymer Blend Films

To translate our understanding of the photolysis kinetics in bulk samples to patterned WEL samples, UV–vis absorbance spectroscopy functionality was built into the fabrication setup, enabling waveguide formation to be monitored in situ (see Section 3.2 Supporting Information for further details). The ability to monitor the progress of polymerization is particularly advantageous, as WEL formation is likely affected by the incorporation of a luminophore. In situ monitoring also allows for the systematic and reproducible fabrication of samples using spectroscopic events as reference points rather than a simple timer. A collimated light source with a higher irradiance (>1 mW cm^–2^) was required for waveguide channel formation to be observed, primarily due to the implementation of the chrome photomask, which reduced the incident irradiance upon the sol by half (Figure S1, Supporting Information). As such, the halogen lamp used in the previous studies was replaced with a collimated white LED with adjustable irradiances up to 2.6 mW cm^–2^.

**Figure 5:**
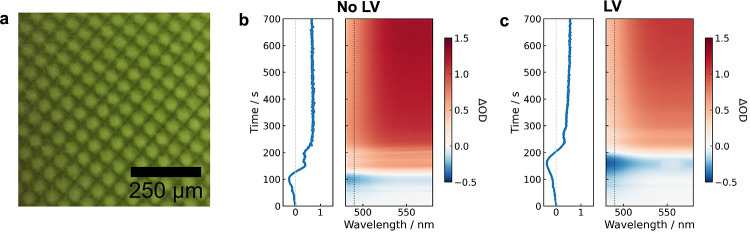
In situ monitoring of LWEL formation by UV–vis spectroscopy. (a) Micrograph of LWEL film (**Blend 3:2**) with 40 μm channels and **LV** incorporated as a luminophore. In situ spectral map of **Blend 3:2** (b) without **LV** and (c) with **LV** as polymerization proceeds. The depletion of Omnirad 784 is evident in (b, c) from the negative ΔOD at early times (<200 s) below λ = 500 nm. Scattering dominates the change in transmission at later times (>200 s), manifesting as an increase in ΔOD across the entire wavelength range (480–580 nm).

Polymer blends consisting of 3 parts **Acr-Sil** to 2 parts **Epo** by volume (herein referred to as **Blend 3:2**), either with (0.02 wt %) or without **LV**, were prepared and injected into 3D-printed sample cells. The 3:2 composition was chosen because it had a lower amount of glass adhering **Epo** component than a 1:1 blend, making it easier to separate WELs from glass slides. Samples were loaded into the fabrication setup (see Figure S1, Supporting Information) and illuminated by the white LED beam for 10 min at an irradiance of 1.4 mW cm^–2^. A representative micrograph of the obtained LWEL films is shown in Figure 5a. We note that there was no measurable difference in the channel size between WELs made with and without **LV**.

The change in optical density (ΔOD) of LWEL films during photopolymerization was monitored with irradiation time. Monitoring was carried out across the wavelength range of 480–580 nm via in situ absorbance spectroscopy. Over this wavelength range, both the consumption of Omnirad 784 and the formation of waveguide channels were detectable. In the absence of **LV**, the spectral map shows an initial induction period of ∼100 s before any significant changes in transmission are observed (Figure 5b). An increase in the transmission is evident below 500 nm after 100 s, consistent with the depletion of Omnirad 784, as observed for the bulk samples. This induction period is related to the change in the probe light geometry used for UV–vis absorbance spectroscopy measurements on bulk and patterned blends. For (L)WEL samples, the excitation and probe source are the same, meaning the excitation irradiance is nonuniform and drops exponentially with the path length. An increase in the transmittance is seen when enough Omnirad 784 is depleted across the path length such that light exits the sol and reaches the spectrometer. After 200 s, scattering losses become more pronounced, manifested as an increase in optical density (OD). We attribute the scattering losses to the formation of waveguide channels, as this behavior was not observed in blends without waveguide patterning.

The addition of **LV** shifts the peak in Omnirad 784 consumption from *t* = 100 to 150 s and results in more Omnirad 784 being consumed (apparent from the more considerable increase in transmission, Figure 5c), as seen in bulk samples. Furthermore, the increased scattering at later times is less pronounced upon the addition of **LV**, which may be indicative of slight changes in refractive index contrast between the waveguide channels and their surroundings induced by the photoinitiator-luminophore interaction.

**Figure 6:**
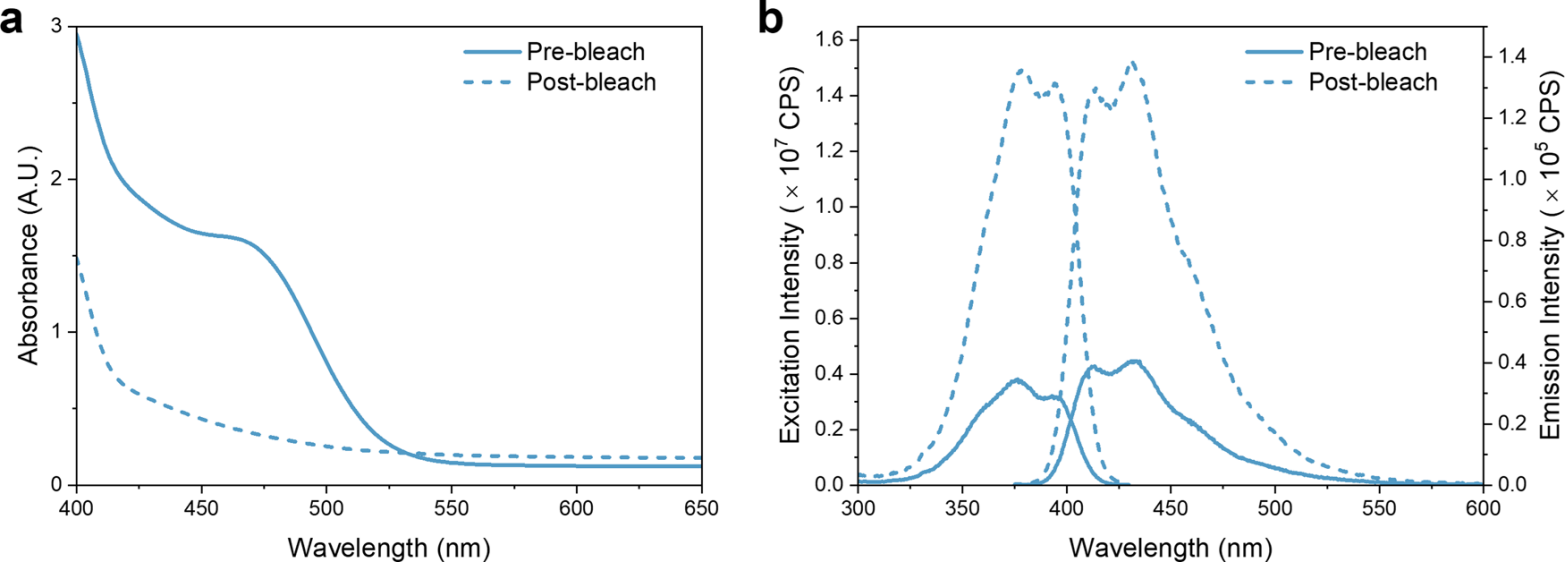
Use of photobleaching to eliminate unused photoinitiators in LWELs (3:2 blend). (a) UV–vis absorbance spectra of LWELs pre- and postbleach, with **LV** as the luminophore. (b) Corresponding excitation (λ_em_ = 440 nm) and emission (λ_exc_ = 365 nm) spectra of an LWEL sample.

The in situ absorbance measurements also reveal that not all photoinitiators are consumed during the reaction. Remaining Omnirad 784 was removed by irradiating the sample with a white LED (2.5 min at 53 mW cm^–2^ irradiance) in conjunction with a long pass filter (495 nm) to prevent luminophore photodegradation. Our results show the disappearance of the Omnirad 784 shoulder (see Figure 6a) postbleaching and a 4-fold increase in fluorescence counts (Figure 6b). We note that control measurements on a sample without LV exhibited negligible fluorescence over the same wavelength range, which excludes Omnirad 784 as a source of fluorescence. Hence, the observed increase in fluorescence intensity is accounted for by the decrease in parasitic absorption from Omnirad 784 and a decrease in electron transfer from the luminophore to the photoinitiator.

The emission decay behavior of **LV** in LWELs pre- and postbleaching was also investigated. In contrast to the earlier studies in solution, the decay curves were best modeled by a double-exponential decay, indicating the presence of two distinct luminophore environments (see Table S5, Supporting Information). The major component has a lifetime, τ_1_ ∼ 5 ns (f_1_ ∼ 90%), that is comparable to the natural lifetime of **LV** in toluene at low concentration. The secondary component has a lifetime τ_2_ ∼ 2 ns (f_2_ ∼ 10%), which is similarly in excellent agreement to the quenched lifetime of **LV** in toluene at high concentration in the presence of Omnirad 784/OPPI. In contrast to the solution, in the solid LWEL films, diffusion is negligible on the time scale of this measurement, enabling both contributions to be resolved. Interestingly, f_2_ decreases to 5.0% following photobleaching, confirming the luminophore-photoinitiator interaction observed previously, which disappears as the photoinitiator is depleted. As the interaction between Omnirad 784 and **LV** is reduced upon bleaching, this leads to a higher fraction of emissive luminophore species, further supporting the increased fluorescence intensity of the LWEL samples upon photoinitiator bleaching.

## Conclusions

The relationship between luminophore-photoinitiator interactions and the photolysis kinetics during photopolymerization of methacrylate-siloxane and epoxide homopolymers and their blends as model systems has been examined. Initial rate analysis of the photoinitiator consumption revealed differences in the photolysis rates for all bulk polymer systems in the absence and presence of **LV**, with a notable (∼40%) rate increase observed for **Blend 1:1**. Interpretation of these rates is highly complex since **LV**, Omnirad 784, and OPPI all compete for photon absorption in the emission region of the irradiation source, to an extent that is determined by the relative concentration and absorbance coefficient of each species and the photon flux at a given wavelength. However, fluorescence lifetime studies revealed that Omnirad 784 is the primary quenching species in this system, with DFT calculations supporting the transfer of electrons from photoexcited **LV** to Omnirad 784 as the probable quenching process. This pathway provides a secondary mechanism by which the photoinitiator free-radical species may be generated, leading to an acceleration of the photolysis rate and an increased degree of consumption of the photoinitiator. However, as the rate of the electron transfer process will be highly dependent on the proximity of the two species, the absolute concentration and fluidity of the medium are also important. In situ UV–vis absorbance spectroscopy to monitor Omnirad 784 consumption during WEL formation further indicates that **LV**-photoinitiator interactions determine the photopolymerization kinetics and provides an additional method to confirm channel formation through the observation of scattering. Notably, the results demonstrate that the photoinitiator is not completely consumed by the time the WEL channels are formed. Retention of the photoinitiator within the final LWEL is highly undesirable as it may lead to photodegradation of the luminophore during use. We have shown that the unconsumed photoinitiator can be removed using a simple postfabrication irradiation protocol, which further leads to a fluorescence enhancement as the electron transfer pathways are eliminated.

Overall, our studies of these model photopolymer systems show that both understanding and quantifying the extent of luminophore-photoinitiator interactions are crucial to the fabrication of high-quality bulk and micropatterned luminescent polymers. The addition of a luminophore influences the photopolymerization kinetics in an unpredictable way, which must be considered in conjunction with the absorption efficiency of each component against the wavelength-dependent emission of the irradiation source when designing new photocurable polymer formulations. For micropatterned systems, such as WELs, this is of critical importance since incomplete or inhomogeneous polymerization may influence the integrity of the waveguide channels, for example, due to variation in density or refractive index contrast, which would be highly detrimental for light capture or conversion applications. Future manufacture of WELs will thus benefit significantly from a real-time optimization approach using in situ spectroscopic monitoring.

## Supporting Information Available

Materials, precursor sol preparation, photopolymerization conditions, spectroscopic characterization, computational simulations, polymerization mechanisms, photon absorbance of sol components, photolysis kinetics, initiation yields, and fluorescence decay curves (PDF)
