# Supplementary material for: Interplay of Luminophores and Photoinitiators during Synthesis of Bulk and Patterned Luminescent Photopolymer Blends
Source: ACS Appl Polym Mater. 2024 May 27;6(11):6314–22. doi: 10.1021/acsapm.4c00484 (PMC11186006; doi:10.1021/acsapm.4c00484)
Supplement: Supplementary file 1 — ap4c00484_si_001.pdf [file ap4c00484_si_001.pdf]

SUPPORTING INFORMATION

Interplay of Luminophores and Photoinitiators during Synthesis of Bulk and Patterned Luminescent Photopolymer Blends

Helen Tunstall-García,[1,†] Takashi Lawson,[1,†] Kathryn A. Benincasa,[2] Andrew W. Prentice,[3] Kalaichelvi Saravanamuttu[2] and Rachel C. Evans[1*]

[1] *Department of Materials Science and Metallurgy, University of Cambridge, Cambridge, CB3 0FS, UK.*
[2] *Department of Chemistry and Chemical Biology, McMaster University, Hamilton, L8S 4M1, Canada.*
[3] *School of Engineering & Physical Sciences, Heriot-Watt University, Edinburgh, EH14 4AS, UK.*

* Corresponding Author: Rachel C. Evans (rce26@cam.ac.uk)
† These authors contributed equally to this work

Table of Contents

1 MATERIALS **2**
2 PRECURSOR SOL PREPARATION **2**
2.1 PREPARATION OF METHACRYLATE-SUBSTITUTED SILOXANE SOL 2
2.2 PREPARATION OF MULTICOMPONENT EPOXIDE SOL 2
2.3 PREPARATION OF POLYMER BLENDS 2
3 PHOTOPOLYMERIZATION CONDITIONS **3**
3.1 BULK HOMOPOLYMERS AND BLENDS 3
3.2 OPTICAL SETUP FOR LWEL FILM FABRICATION 3
3.3 REMOVAL OF UNREACTED PHOTOINITIATOR 4
4 SPECTROSCOPIC CHARACTERIZATION **4**
4.1 UV-VIS ABSORBANCE SPECTROSCOPY 4
4.2 STEADY-STATE PHOTOLUMINESCENCE (PL) SPECTROSCOPY 4
4.3 TIME-RESOLVED EMISSION SPECTROSCOPY 4
4.4 TIME-DEPENDENT ABSORBANCE SPECTROSCOPY TO MONITOR PHOTOPOLYMERIZATION 4
5 COMPUTATIONAL SIMULATIONS **5**
6 POLYMERIZATION MECHANISMS **5**
7 SUPPLEMENTARY EXPERIMENTAL DATA **7**
7.1 PHOTOPOLYMERIZATION OF COMPONENT EPOXIDE SOL USING OMNIRAD™ 784 7
7.2 PHOTON ABSORPTION FOR SOL COMPONENTS UNDER DIFFERENT EXCITATION SOURCES 7
7.3 PHOTOLYSIS KINETICS IN BULK HOMOPOLYMERS AND THEIR BLENDS 8
7.4 CALCULATION OF THE INITIATION YIELD 10
7.5 TIME-RESOLVED FLUORESCENCE QUENCHING STUDIES ON LV SOLUTIONS 11
7.6 COMPUTATIONAL RESULTS 11
7.7 PHOTOBLEACHING OF THE PHOTOINITIATOR FROM LWELS 14
8 REFERENCES **16**

1 Materials

All chemicals and reagents were purchased from commercial suppliers and used as received. 3-(trimethoxysilyl)propyl methacrylate (MAPTMS, 98%), hydrochloric acid (HCl, ACS reagent grade, 37%), 3,4-epoxycyclohexylmethyl 3,4–epoxycyclohexanecarboxylate (EEC, 90%) and poly(tetrahydrofuran) (pTHF, M_n = 250 g mol^{-1}) were purchased from Sigma Aldrich. Epoxypropoxypropyl terminated polydimethylsiloxane (pPDMS, M_w = 363 g/mol, 90%) was purchased from Gelest Inc. The photoinitiator [(bis(η^5-cyclopentadienyl) bis(2,6-difluoro-3-(1H-pyrrole-yl)-phenyl) titanium (IV)] (Omnirad™ 784, 99%) was purchased from IGM Resins. The co-initiator 4-octyloxyphenyl phenyliodonium hexafluoroantimonate (OPPI) was kindly donated by Hampford Research Inc. The luminophore 2-(2-ethylhexyl)-6,7-dimethoxy-1*H*-benzo[de]isoquinoline-1,3(2*H*)-dione (Lumogen® Violet) was procured from BOC Sciences.

2 Precursor Sol Preparation

2.1 Preparation of methacrylate-substituted siloxane sol

Methacrylate-substituted siloxane sols were prepared through two-step acid-catalyzed hydrolysis and condensation of MAPTMS. First, 0.05 M HCl (0.30 g) was added to MAPTMS (4.89 g, 0.0197 moles) yielding a two-phase mixture which transformed into a homogenous, transparent, colorless liquid after stirring at room temperature (RT) for 15-20 min (Sol 1, Figure 1 – main paper). The titanocene-based free-radical photoinitiator Omnirad™ 784 was then added to the sol (0.5 wt%), along with Lumogen® Violet (0.02 wt%) if using. The mixture was protected from ambient light with aluminum foil wrapping and left to stir for six days at room temperature (RT).

2.2 Preparation of multicomponent epoxide sol

The epoxide sol (Sol 2, Figure 1, main paper) was prepared following a previously reported method with the photoinitiator (camphorquinone) substituted by Omnirad™ 784 and the proton donor (benzyl alcohol) substituted by pTHF.[1] EEC (51 wt%), pPDMS (26 wt%), pTHF (20 wt%) Omnirad™ 784 (0.2 wt%) and OPPI (2.0 wt%) were mixed in a single pot until homogeneous. Lumogen® Violet was added at a 0.02 wt% loading if using. The mixture was protected from ambient light with aluminum foil wrapping and left to stir at room temperature for at least 48 hours before use.

2.3 Preparation of polymer blends

Precursor blends were created by mixing the methacrylate-substituted siloxane sol and the component epoxide sol at the required ratio under stirring for 30 min at RT to ensure homogenous mixing. Herein, references to the composition of the photosensitive blends will be made by the volume ratio of the amount of acrylate to epoxide (*i.e.*, blend 3:2 refers to a formulation with 3 parts methacrylate-substituted siloxane to 2 parts epoxide).

3 Photopolymerization Conditions

3.1 Bulk homopolymers and blends

Bulk polymer samples were fabricated by vertical irradiation of the liquid sol contained in an Eppendorf UVette® with an MRL-58 multiple ray lamp, with a cool white halogen tube as the light source (0.63 mW cm^{-2}) for an irradiation period between 10 to 30 minutes. Samples appeared solid after irradiation with partial discoloration due to photobleaching of Omnirad™ 784.

3.2 Optical setup for LWEL film fabrication

To fabricate LWEL films, an optical setup was built with the ability to monitor the fabrication process *in situ* by UV-Vis absorbance spectroscopy (see Figure S1). All optical components were mounted on a dovetail rail (Thorlabs RLA600/M). A white LED (Thorlabs MCWLH7) was collimated by a lens (f = 32 mm, Thorlabs SM2F32-A), and the size of the illuminated region was controlled using an adjustable iris (Thorlabs ID50/M). An LED driver (Thorlabs LEDD1B) enabled the irradiance to be adjusted up to 2.6 mW cm^{-2}. A custom chrome photomask (Micro Lithography Services) with a 40 μm by 40 μm grid pattern (Λ = 80 μm) was used to block light from propagating in certain regions of the sample cell. Transmitted light was then collimated by a lens (f = 150 mm, Thorlabs AC508-150-A-ML) and 50/50 beamsplitter cube (Thorlabs BS031) used to capture an image with its associated spectrum simultaneously. A lens (f = 75 mm, Thorlabs AC508-075-A-ML) was used to focus the light onto a CMOS chip (Thorlabs Zelux 1.6 MP) allowing live experimental capture. A fiber collimation package (f = 7.86 mm, Thorlabs F240SMA-532) was used to transport light to a spectrometer (Ocean Optics Flame). Neutral density filters (Thorlabs NE2R0XA series) were used to decrease the power in both the camera and spectrometer. A Python script was written to interface with the spectrometer and CMOS chip, enabling time-stamped images and spectra to be collected regularly.

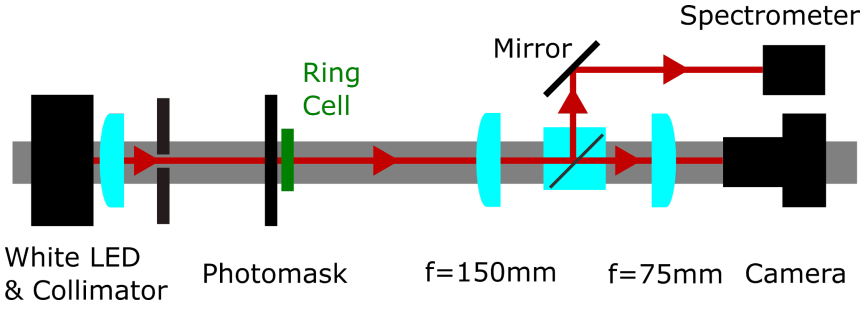

Figure S1 | Schematic of the LWEL fabrication and characterization set-up. Transmitted light through the LWEL sample can simultaneously be analyzed by a camera and its spectrum by a spectrometer to monitor changes in absorbance *in situ*.

The precursor sol (homopolymer or blend) was injected into a custom-designed sample cell consisting of a 3D-printed thermoplastic polyurethane (TPU) spacer sandwiched between two glass slides. The TPU spacer was printed to a thickness of 1 mm with an Ultimaker3 3D printer. Silicon sealant was used to form a liquid-tight seal with glass slides. Samples were irradiated by a collimated White LED beam for 10 minutes with an irradiance behind the mask of 1.4 mW cm^{-2} (measured by a Thorlabs S120VC photodiode). On removal, samples were solid and appeared hazy due to waveguide lattice formation.

3.3 Removal of unreacted photoinitiator

Unreacted Omnirad™ 784 was removed from LWEL films by irradiating with a high-power white LED array (Brennenstuhl JARO 7003 M, irradiance = 53 mW cm^{-2}). LWEL films were covered with a long pass filter (495 nm) and irradiated for 2.5 minutes.

4 Spectroscopic Characterization

4.1 UV-Vis absorbance spectroscopy

UV-Vis absorbance spectra were recorded on a Perkin Elmer Lambda 750 spectrophotometer. The spectra were collected using a wavelength scan method with a slit width of 2 nm and a step size of 1 nm. For solution samples, spectra were collected using a 10 mm quartz cuvette (Hellma) and measured against a solvent reference. For solid (bulk) samples, spectra were measured against air as the reference.

4.2 Steady-state photoluminescence (PL) spectroscopy

A Horiba Jobin Yvon Fluorolog-3 fluorescence spectrometer was used to record photoluminescence excitation and emission spectra. The entrance and exit slits were adjusted to obtain a maximum PL intensity in the region of interest while maintaining it within the area of linear response. The integration time was 0.1 s. Emission spectra were corrected using the radiometric correction factors supplied by the manufacturer.

4.3 Time-resolved emission spectroscopy

Photoluminescence decay measurements were performed using the time-correlated single photon counting method (TCSPC) on an FLS1000 PL spectrometer (Edinburgh Instruments). The excitation source was a pulsed laser of wavelength 375 nm (EPL-375, pulse width <100 ps, pulse frequency 20 MHz). The emission decay was recorded at 430 nm using a high-speed photomultiplier tube (HS-PMT-920, 200 ps response time) or visible photomultiplier tube (PMT-980, 600 ps response time) with TCC2 counting electronics. Deconvolution and data-fitting were performed as individual fits to each emission decay using a single or double exponential decay function from 5-50 ns using the FAST software package (Edinburgh Instruments). The fit quality was assessed using reduced chi-square statistics, χ^2, and the statistical randomness of the residuals.

4.4 Time-dependent absorbance spectroscopy to monitor photopolymerization

To monitor the reaction kinetics, the consumption of the photoinitiator Omnirad™ 784 was measured by time-resolved UV-Vis absorbance spectroscopy. Unirradiated components were loaded into an Eppendorf UVette® (2 mm path length) with a transmission range between 220 - 1600 nm and subjected to irradiation from above. Spectra were collected at 30-second intervals using an Ocean Optics Flame spectrometer, together with an Ocean Optics DH-2000 white light source, and the absorbance was converted to a concentration by the Beer-Lambert law. The concentration was calculated from the average absorbance in the wavelength range of 450 nm to 455 nm, coinciding with one of the absorbance maxima of Omnirad™ 784.

5 Computational simulations

Density functional theory (DFT)[2,3] calculations were performed using Orca 5.0.[4,5] All ground-state structures were confirmed to be minimum-energy arrangements via analytic hessian computations, displaying positive curvature along all vibrational modes. The excited states were modelled by linear response time-dependent (TD) DFT,[6] with the nature of the stationary point validated by numerical hessian computations (± 5×10^{-3} Bohr increments). The hybrid exchange-correlation functional B3LYP[7–9] was used in conjunction with the Karlsruhe valence triple-ζ polarized basis set, def2-TZVP.[10,11] The CAM-B3LYP[12] functional was also tested and was found to give a poorer agreement to experiment (see Figure S7). The external reaction field was modelled by the inclusion of a dielectric continuum model, specifically the conductor-like polarizable continuum model (CPCM).[13] A dielectric constant of 2.4 (toluene) was used. The adiabatic reaction energies are given with respect to electronic (E) and free energies (G), the later calculated through the standard ideal gas, rigid rotor, and harmonic oscillator statistical models and at a temperature of 298.15 K.

6 Polymerization Mechanisms

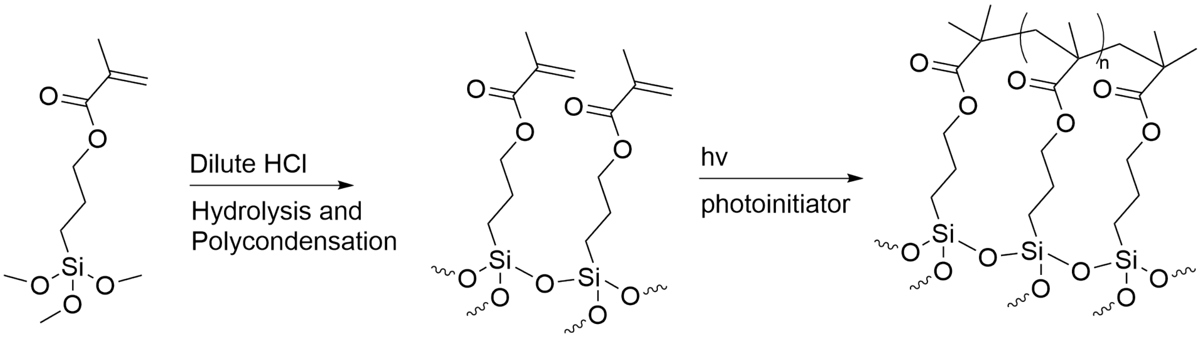

Scheme S1 I Polymerization mechanism for MAPTMS (Sol 1) consisting of an initial acid-catalyzed hydrolysis-condensation reaction to form the siloxane network, followed by photoinitiated free-radical polymerization of the methacrylate groups.

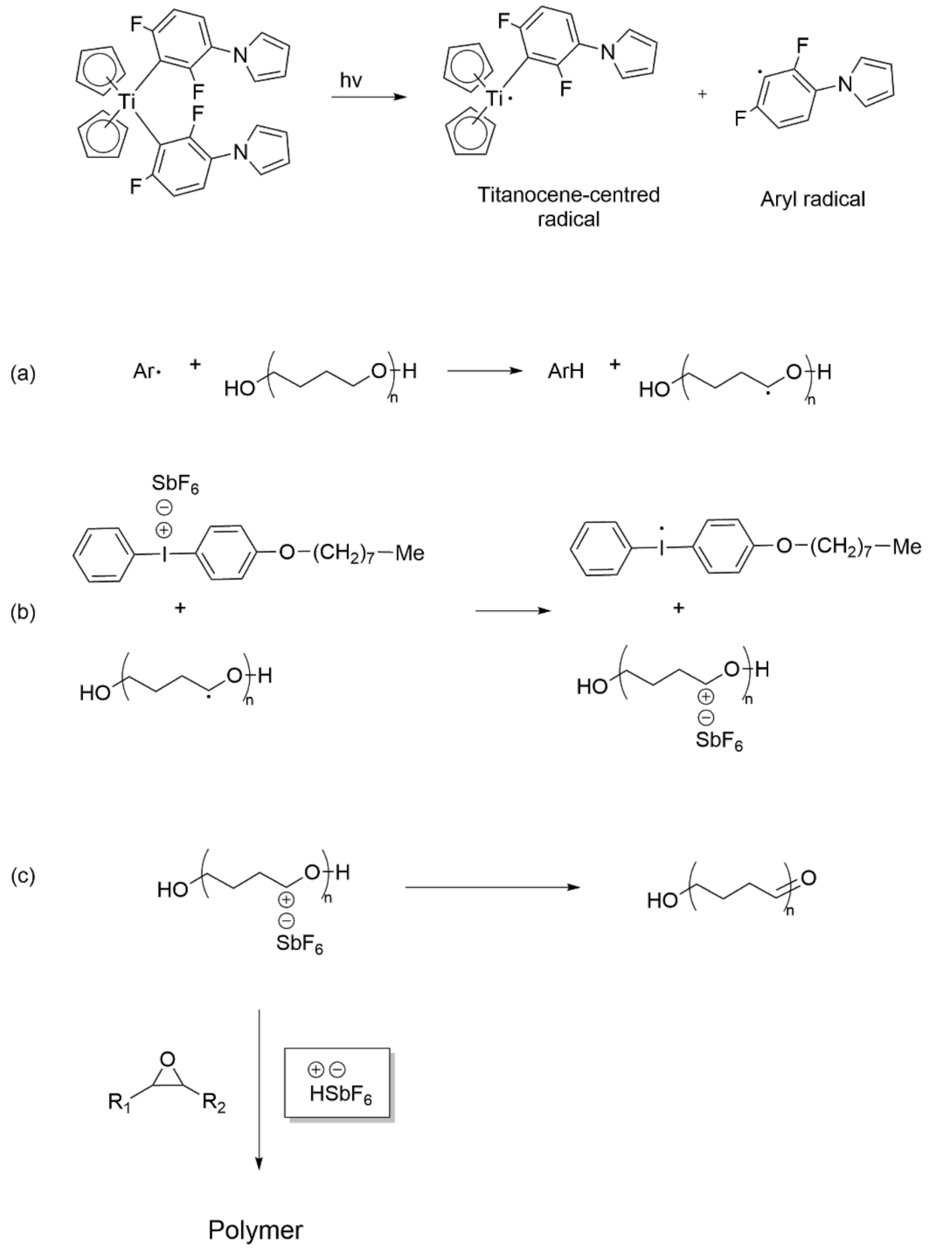

Scheme S2 I Free-radical promoted cationic polymerization mechanism for component epoxide sol (Sol 2). (a) Irradiation of Omnirad™ 784 results in forming aryl- and titanium-centered radicals. The polyTHF hydrogen donor undergoes hydrogen-abstraction by the aryl radical. (b) The resulting α-hydroxyalcohol radical reacts with the OPPI aryliodonium to give an iodine-centered radical and an α-hydroxyalcohol cation. (c) The α-hydroxyalcohol cation spontaneously deprotonates, resulting in an aldehyde and a hexafluoroantimonate superacid capable of initiating epoxide polymerization. All radical species generated throughout the process can catalyze the photoinitiation reaction.

7 Supplementary Experimental Data

7.1 Photopolymerization of component epoxide sol using Omnirad™ 784

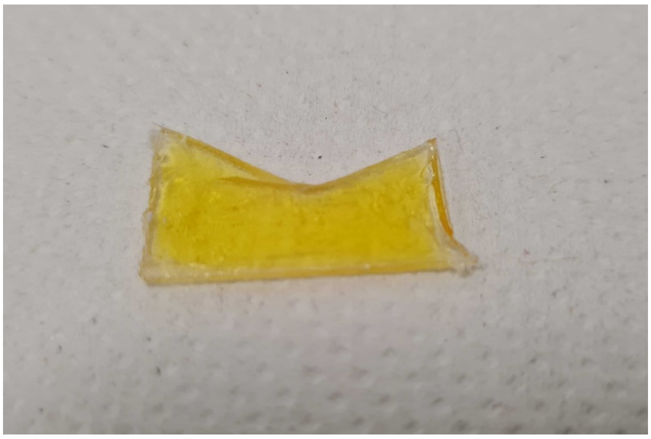

Figure S2 I Photograph of an epoxide WEL sample fabricated using Omnirad™ 784 (0.05 g) as the photoinitiator, with OPPI as the co-initiator, under irradiation for 12 minutes (irradiance = 1.5 mW cm^{-2}, white LED Thorlabs MCWLH7) The longest side is 2.5 cm in length. The epoxide sol consisted of EEC (2.54 g), pPDMS (1.31 g), pTHF (1.00 g), OPPI (0.10 g) and Omnirad™ 784 (0.05 g).

7.2 Photon absorption for sol components under different excitation sources

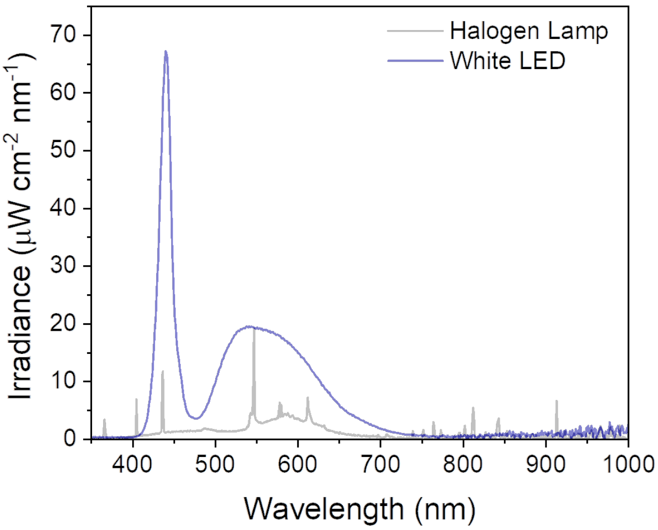

Figure S3 I Emission spectra of the two light sources used for photopolymerization: MRL-58 halogen lamp (bulk samples) and Thorlabs MCWLH7 white LED (LWELs). Spectra were obtained using a calibrated NIST-traceable ILT950 spectroradiometer with W2 wide-eye diffuser. The integrated irradiance in the visible region (380 - 780 nm) was 0.63 mW cm^{-2} for the cool white halogen source and 1.4 mW cm^{-2} for the white LED. The background lab lighting irradiance was 0.05 mW cm^{-2}.

Table S1 | Number of photons absorbed by different species in toluene under different excitation sources (halogen and white LED), using the same loadings for each component as in the sols.

Sol	Component	Loading (wt%)	Loading (mM)	LVRPA[a] with Halogen Lamp (μM s^{-1})	LVRPA[a] with White LED (μM s^{-1})
Acr-Sil	Omnirad™ 784	0.49	10	0.97 (66%)	8.87 (74%)
	LV	0.020	0.59	0.50 (34%)	3.18 (26%)
Epo	Omnirad™ 784	0.10	4.1	0.81 (54%)	7.74 (64%)
	OPPI	2.0	34	0.18 (12%)	1.18 (10%)
	LV	0.020	0.59	0.50 (34%)	3.18 (26%)
Blend 1:1	Omnirad™ 784	0.35	7.0	0.90 (60%)	8.39 (69%)
	OPPI	1.0	17	0.10 (7%)	0.61 (5%)
	LV	0.020	0.59	0.50 (33%)	3.18 (26%)

[a]*LVRPA = local volumetric rate of photon absorption. Percentages give the fraction of photons absorbed by that component.*

7.3 Photolysis kinetics in bulk homopolymers and their blends

The photolysis kinetics during photopolymerization of bulk **Acr-Sil, Epo** and **Blend 1:1** sols were studied by following the consumption of Omnirad™ 784 by time-resolved UV-Vis absorbance spectroscopy. **Acr-Sil** sols showed a considerable increase in scattering as the polymerization proceeded, manifesting as a linear absorbance feature above 550 nm (Figure S4a). We attribute this feature to the formation of micron-sized nuclei as the addition polymerization of methacrylate groups proceeds. To correct for scattering, the **Acr-Sil** spectra were subtracted by the average absorbance in the wavelength range 575-600 nm, where no absorbance change due to Omnirad™ 784 consumption is expected (Figure S4b). This scattering correction was unnecessary for **Epo** and **Blend 1:1** samples.

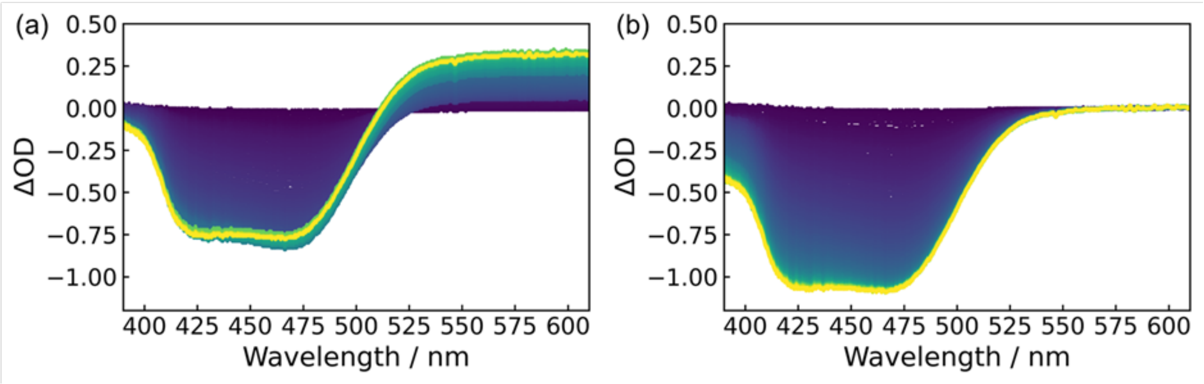

Figure S4 I (a) Plot of the raw absorbance data of an **Acr-Sil** (LV) sol during photolysis of Omnirad™ 784 showing a clear scattering artefact above 550 nm. (b) Plot of the data corrected for scattering by subtracting the average absorbance in the wavelength range 575 nm to 600 nm from all data points in (a).

Molar absorbance coefficients, ε, for Omnirad™ 784 in each sol were determined using a known photoinitiator concentration using the average absorbance over the wavelength range 450-455 nm. This wavelength range was selected to ensure Omnirad™ 784 as the sole absorbing species while maintaining a high absorbance coefficient. The values obtained were: $\varepsilon_{\text{Acr-Sil}}$ = 711 M^{-1} cm^{-1}, ε_{Epo} = 1,213 M^{-1} cm^{-1} and $\varepsilon_{\text{Blend(1:1)}}$ = 893 M^{-1} cm^{-1} (see Figure S5). Once the molar absorbance coefficients were calculated, the molar degree of consumption of Omnirad™ 784 could be computed from time-dependent UV-Vis absorbance spectra. The obtained concentration profiles were also normalized to the starting concentration of Omnirad™ 784 to give a conversion (%) profile (see Table 1, main paper).

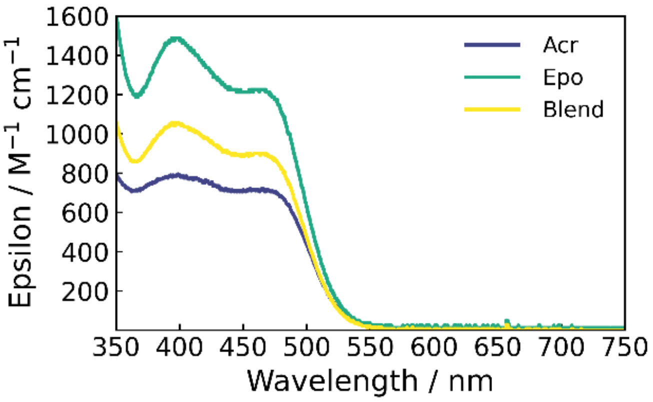

Figure S5 | (a) UV-Vis absorbance spectra of polymer sols with and without Omnirad™ 784. Absorbance values were averaged in the wavelength range 450 nm to 455 nm then converted to an absorbance coefficient in (b). Omnirad™ 784 concentration in acrylate (Acr-Sil) sol was 10 mM, epoxide (Epo) sol was 4 mM, and blend sol was 7 mM. The path length was 2 mm. The absorbance coefficients of Omnirad™ 784 in each sol were calculated to be: Acr-Sil 711 M^{-1} cm^{-1}, Epo 1,213 M^{-1} cm^{-1} and Blend 893 M^{-1} cm^{-1}.

The initial rate of photolysis for the different sols was calculated from the slope of the linear curve of the molar consumption of Omnirad™ 784 in the range of 0-150 sec (see Figure 3, Table 1, main paper), according to a *pseudo*-zero-order reaction rate. Further tests confirmed that under identical irradiation conditions, at a lower Omnirad™ 784 concentration (0.05 wt%,

1 mM), the photoinitiator concentration limits the reaction and proceeds under pseudo-first-order conditions (see Figure S6).

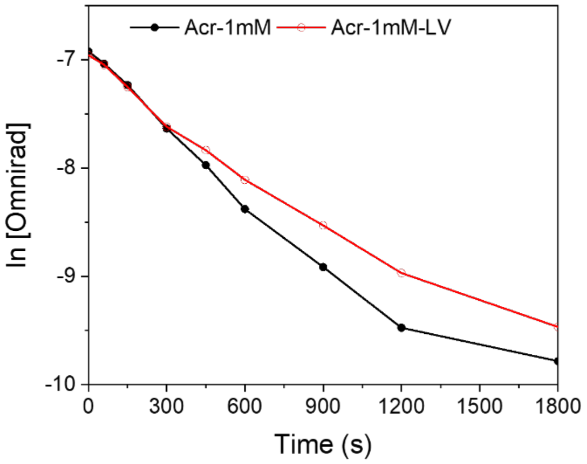

Figure S6 I First-order kinetic decay plot of the photolysis of Omnirad™ 784 present at low concentration (1 mM) in **Acr-Sil** sols upon irradiation with a halogen lamp, showing a linear relationship between [Omnirad] and time.

7.4 Calculation of the initiation yield

The initiation yield is the moles of Omnirad™ 784 consumed per mole of photons absorbed by Omnirad™ 784. The moles of incident photons (N_{incident}) can be calculated based on spectral power (I) and wavelength (λ):

$$N_{\mathrm{incident}} = \frac{I}{E_{ph}} = \frac{I\,\lambda}{N_A hc} \qquad \textit{Eq 1}$$

The local volumetric rate of photon absorption (LVRPA) can then be calculated based on N_{incident} and the absorbance of Omnirad™ 784 (Abs).

$$\mathrm{LVRPA} = N\left(1 - 10^{-\mathrm{Abs}}\right) = \frac{1}{N_A hc}\lambda\left(1 - 10^{-\mathrm{Abs}}\right)I \qquad \textit{Eq 2}$$

where Abs can be calculated using the wavelength-dependent absorbance coefficient ($\varepsilon(\lambda)$), concentration (c) and path length (l), *i.e.* Beer-Lambert law:

$$\mathrm{Abs} = \varepsilon(\lambda)cl \qquad \textit{Eq 3}$$

For a broadband lamp spectrum, we sum over all emitted wavelengths:

$$\mathrm{LVRPA} = \frac{1}{N_A hc}\sum_{\lambda}\lambda(1 - 10^{-\mathrm{Abs}(\lambda)})I(\lambda) \qquad \textit{Eq 4}$$

The lamp or LED spectra ($I(\lambda)$) used in this calculation is shown in Figure S3. The absorbance coefficients ($\varepsilon(\lambda)$) used in this calculation are shown in Figure 2 (main text)**.** The absorbance was calculated with a path length (l) of 1 cm and the starting concentration of Omnirad™ 784 (c) in each sol (Table S1).

The initiation yield (Y) is then given by the Omnirad™ 784 consumption rate (k) divided by the LVRPA.

$$Y = \frac{k}{\mathrm{LVRPA}} \quad \text{Eq 5}$$

7.5 Time-resolved fluorescence quenching studies on LV solutions

Time-resolved fluorescence decay curves were fitted to an exponential decay model:

$$I(t) = \sum_{i}^{n} \alpha_i \exp\left(-t/\tau_i\right) \quad \text{Eq 6}$$

where $I(t)$ is the photoluminescence intensity as a function of time, t, normalized to the intensity at $t = 0$, α_i is the pre-exponential factor of the component and τ_i is the lifetime of the i_{th} decay component. The fractional contribution is given by:

$$f_i = \frac{\alpha_i \tau_i}{\sum_j^n \alpha_j \tau_j} \quad \text{Eq 7}$$

For LV solutions in toluene containing no quencher, Omnirad™ 784 or OPPI, a single exponential decay (n = 1) yielded a good fit. For LWEL samples, pre- and post-bleach (see Section 7.7), a bi-exponential decay (n = 2) was required. The quality of the fit was evaluated by the χ^2 value and the randomness of the residuals.

Table S2 | Lifetimes obtained from single-exponential fits to decay curves for LV solutions in toluene in the absence and presence of different quenchers. All decay curves are well-described by a single exponential decay. Decay curves obtained with excitation at 375 nm and emission at 430 nm.

Sample	τ (ns)	$\Delta\tau$ (ns)	χ^2
LV (5 µM)	4.646	0.0001	1.088
LV (5 µM) + Omnirad™ (200 µM)	4.612	0.0001	1.076
LV (5 µM) + OPPI (550 µM)	4.577	0.0001	1.104
LV (300 µM)	5.096	0.0002	1.239
LV (300 µM) + Omnirad™ (10 mM)	2.252	0.0007	1.214
LV (300 µM) + OPPI (30 mM)	3.669	0.0003	1.160
LV (300 µM) + Omnirad™ (10 mM) + OPPI (30 mM)	1.975	0.0009	1.274

7.6 Computational Results

The B3LYP functional was selected as it gave better agreement to experimental spectra (Figure S7). The broad absorption band of Omnirad™ 784, located between approximately 370 and 480 nm, consists of three states, S1 (448.6 nm), S5 (416.5 nm) and S7 (385.1 nm), all with oscillator strengths marginally greater than 0.01 and are of ligand-to-metal charge

transfer character. On the other hand, the Lumogen® Violet spectrum is dominated by a single peak, S1 (372.6 nm), with an oscillator strength of 0.41 and is of ππ* character.

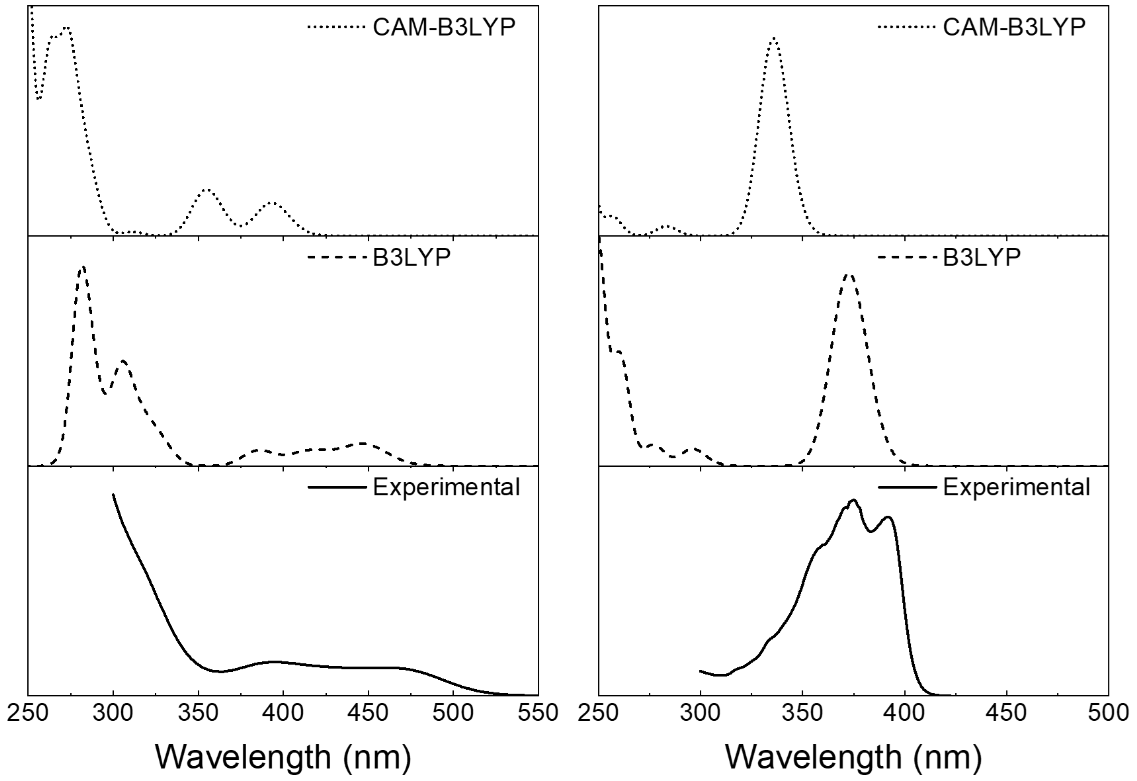

Figure S7 | Comparison of the CAM-B3LYP, B3LYP and experimental absorbance spectra of a) Omnirad™ 784 and b) Lumogen Violet®, with the B3LYP functional showing a better overlap with the experimental results.

Following Kasha's rule, the electron transfer reaction energetics were computed with respect to the S1 state of either Omnirad™ 784 or Lumogen® Violet. The feasibility of electron transfer reactions was investigated from a thermodynamic viewpoint under both adiabatic and vertical transition conditions, as well as through the analysis of the HOMO-LUMO energies of Omnirad™ 784 and LV.

Under adiabatic conditions (Table S3) all electron transfer processes are endergonic; however, the endergonicity is much lower for reactions involving electron transfer from LV to Omnirad™ 784 (Table S3, entries 1 and 3). Reactions involving photoexcited Omnirad™ 784 are in direct competition with the formation of aryl- and titanium-centered radicals (see Scheme S2) which was found to be extremely exergonic (–223 kJ mol^{-1}).

The vertical transitions, directly after photoexcitation and before the photoexcited species have time to fully relax, were also investigated (Table S4), as this may offset the endergonicity. To mimic this, the free energy correction of the S_0 state was added to the electronic energy of the (vertical) excited state (S_1), which was then used to compute the thermodynamic reaction energy. In this case, the electron transfer from the photoexcited LV($S_{1,vert}$) to the ground state Omnirad™ 784 (S_0) has the lowest endergonicity (1.63 kJ/mol), which is well within the limits of error in typical DFT calculations and could mean that this is a feasible pathway. This is the same reaction of low energy as previously seen (adiabatic calculations, Table S3).

Therefore, from a thermodynamic viewpoint it is likely that the LV acts as the photosensitizer, which is further strengthened by the larger oscillator strengths for the low-energy bands of each species. After photoexcitation, this species would donate an electron to Omnirad™ 784, while photoexcitation of Omnirad™ 784 would likely result in photocleavage.

Table S3 | Adiabatic (ad) reaction energies for electron transfer between LV and Omnirad™ 784.

Reaction	Electronic Energy (kJ/mol)	Free Energy (kJ/mol)
LV($S_{1,ad}$) + Omnirad(S_0) → LV^+ + $Omnirad^-$	32.98	27.42
LV($S_{1,ad}$) + Omnirad(S_0) → LV^- + $Omnirad^+$	123.38	112.27
LV(S_0) + Omnirad($S_{1,ad}$) → LV^+ + $Omnirad^-$	106.13	8.45
LV(S_0) + Omnirad($S_{1,ad}$)→ LV^- + $Omnirad^+$	196.52	93.30

Table S4 | Vertical reaction energies for electron transfer between LV and Omnirad™ 784.

Reaction	Electronic Energy (kJ/mol)	Free Energy (kJ/mol)
**LV($S_{1,vert}$) + Omn(S_0) -> LV^+ + Omn^-**	**19.30**	**1.63**
LV($S_{1,vert}$) + Omn(S_0) -> LV^- + Omn^+	109.69	86.48
LV(S_0) + Omn($S_{1,vert}$) -> LV^+ + Omn^-	73.62	55.95
LV(S_0) + Omn($S_{1,vert}$) -> LV^- + Omn^+	164.01	140.80

Finally, investigation of the HOMO-LUMO energies of Omnirad™ 784 and LV (Figure S8) shows a disfavored electron transfer from the photoexcited Omnirad™ 784 to LV, while electron transfer from the excited LV to the LUMO of the ground state Omnirad™ 784 is favored. This further supports the previous findings that the most likely electron transfer mechanism occurs from an excited-state LV to a ground state Omnirad™ 784.

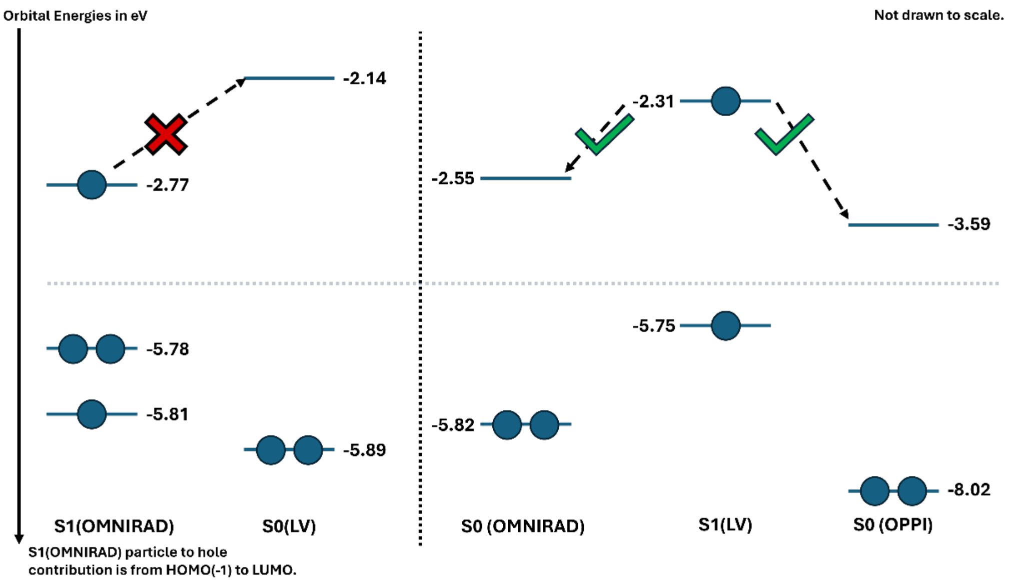

Figure S8 | Diagram of the HOMO-LUMO energies of Omnirad™ 784, OPPI and LV showing the potential electron transfer reactions, and the favored route *via* an excited state LV.

7.7 Photobleaching of the photoinitiator from LWELs

Table S5 | PL decay fitting data (lifetimes, τ_i, and fractional contribution, f_i, for an LV-LWEL sample, pre- and post-bleaching of the unconsumed Omnirad™ 784 photoinitiator. The decay curves (See Figure S9) are well-described by a double exponential decay. Decay curves are obtained with excitation at 375 nm and emission at 430 nm.

Sample	f_1 (%)	τ_1 (ns)	f_2 (%)	τ_2 (ns)	χ^2
LV-LWEL pre-bleach	8.29 ± 0.24	1.99 ± 0.02	91.71 ± 0.72	5.12 ± 0.001	1.036
LV-LWEL post-bleach	5.01 ± 0.21	2.18 ± 0.02	94.97 ± 0.69	5.62 ± 0.001	1.045

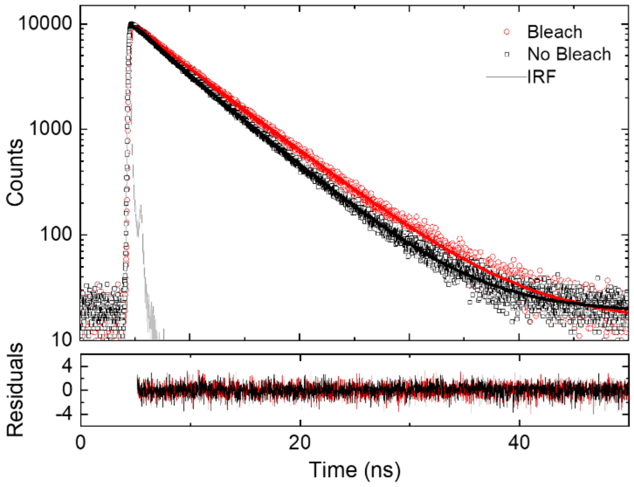

Figure S9 | Photoluminescence decay curves (open symbols) and corresponding fits (solid lines) to a bi-exponential decay function for LV-LWEL (blend 3:2) pre- (black) and post-bleaching (red) of

unconsumed Omnirad™ 784 photoinitiator. Excitation wavelength = 375 nm. Emission wavelength = 430 nm.

8 References

(1) Crivello, J. V. A New Visible Light Sensitive Photoinitiator System for the Cationic Polymerization of Epoxides. *J. Polym. Sci. Part Polym. Chem.* **2009**, *47* (3), 866–875. https://doi.org/10.1002/pola.23203.

(2) Kohn, W.; Sham, L. J. Self-Consistent Equations Including Exchange and Correlation Effects. *Phys. Rev.* **1965**, *140* (4A), A1133–A1138. https://doi.org/10.1103/PhysRev.140.A1133.

(3) Hohenberg, P.; Kohn, W. Inhomogeneous Electron Gas. *Phys. Rev.* **1964**, *136* (3B), B864–B871. https://doi.org/10.1103/PhysRev.136.B864.

(4) Neese, F.; Wennmohs, F.; Becker, U.; Riplinger, C. The ORCA Quantum Chemistry Program Package. *J. Chem. Phys.* **2020**, *152* (22), 224108. https://doi.org/10.1063/5.0004608.

(5) Neese, F. The ORCA Program System. *WIREs Comput. Mol. Sci.* **2012**, *2* (1), 73–78. https://doi.org/10.1002/wcms.81.

(6) Runge, E.; Gross, E. K. U. Density-Functional Theory for Time-Dependent Systems. *Phys. Rev. Lett.* **1984**, *52* (12), 997–1000. https://doi.org/10.1103/PhysRevLett.52.997.

(7) Lee, C.; Yang, W.; Parr, R. G. Development of the Colle-Salvetti Correlation-Energy Formula into a Functional of the Electron Density. *Phys. Rev. B* **1988**, *37* (2), 785–789. https://doi.org/10.1103/PhysRevB.37.785.

(8) Becke, A. D. A New Mixing of Hartree–Fock and Local Density-functional Theories. *J. Chem. Phys.* **1993**, *98* (2), 1372–1377. https://doi.org/10.1063/1.464304.

(9) Stephens, P. J.; Devlin, F. J.; Chabalowski, C. F.; Frisch, M. J. Ab Initio Calculation of Vibrational Absorption and Circular Dichroism Spectra Using Density Functional Force Fields. *J. Phys. Chem.* **1994**, *98* (45), 11623–11627. https://doi.org/10.1021/j100096a001.

(10) Hellweg, A.; Hättig, C.; Höfener, S.; Klopper, W. Optimized Accurate Auxiliary Basis Sets for RI-MP2 and RI-CC2 Calculations for the Atoms Rb to Rn. *Theor. Chem. Acc.* **2007**, *117* (4), 587–597. https://doi.org/10.1007/s00214-007-0250-5.

(11) Weigend, F.; Häser, M.; Patzelt, H.; Ahlrichs, R. RI-MP2: Optimized Auxiliary Basis Sets and Demonstration of Efficiency. *Chem. Phys. Lett.* **1998**, *294* (1), 143–152. https://doi.org/10.1016/S0009-2614(98)00862-8.

(12) Yanai, T.; Tew, D. P.; Handy, N. C. A New Hybrid Exchange–Correlation Functional Using the Coulomb-Attenuating Method (CAM-B3LYP). *Chem. Phys. Lett.* **2004**, *393* (1), 51–57. https://doi.org/10.1016/j.cplett.2004.06.011.

(13) Barone, V.; Cossi, M. Quantum Calculation of Molecular Energies and Energy Gradients in Solution by a Conductor Solvent Model. *J. Phys. Chem. A* **1998**, *102* (11), 1995–2001. https://doi.org/10.1021/jp9716997.
